# DDS and OPC UA Protocol Coexistence Solution in Real-Time and Industry 4.0 Context Using Non-Ideal Infrastructure

**DOI:** 10.3390/s21227760

**Published:** 2021-11-22

**Authors:** Alexandru Ioana, Adrian Korodi

**Affiliations:** Department of Automation and Applied Informatics, Faculty of Automation and Computers, University Politehnica Timișoara, 300223 Timișoara, Romania; tm.alexandru@yahoo.com

**Keywords:** interoperability, Industry 4.0, industrial internet of things, industrial protocols, OPC UA, DDS, publish-subscribe, real-time

## Abstract

Continuing the evolution towards Industry 4.0, the industrial communication protocols represent a significant topic of interest, as real-time data exchange between multiple devices constitute the pillar of Industrial Internet of Things (IIoT) scenarios. Although the legacy protocols are still persistent in the industry, the transition was initiated by the key Industry 4.0 facilitating protocol, the Open Platform Communication Unified Architecture (OPC UA). OPC UA has to reach the envisioned applicability, and it therefore has to consider coexistence with other emerging real-time oriented protocols in the production lines. The Data Distribution Service (DDS) will certainly be present in future architectures in some areas as robots, co-bots, and compact units. The current paper proposes a solution to evaluate the real-time coexistence of OPC UA and DDS protocols, functioning in parallel and in a gateway context. The purpose is to confirm the compatibility and feasibility between the two protocols alongside a general definition of criteria and expectations from an architectural point of view, pointing out advantages and disadvantages in a neutral manner, shaping a comprehensive view of the possibilities. The researched architecture is meant to comply with both performance comparison scenarios and interaction scenarios over a gateway application. Considering the industrial tendencies, the developed solution is applied using non-ideal infrastructures to provide a more feasible and faster applicability in the production lines.

## 1. Introduction

Targeting improvements in availability, safety, productivity, and cost reduction, the manufacturing industry is stepping towards Industry 4.0 and consequently towards the Industrial Internet of Things (IIoT), the two concepts being strongly related. All the improvements within the Industry 4.0 and IIoT context are relying on the ability to collect and exchange data between devices [1,2,3], and therefore on such concepts as interfacing, interoperability, interoperation, and connectivity.

The communication protocols implicitly represent a very important research topic in the current industrial revolution. The first challenge represented by the legacy protocols was overcome by the transition towards the key enabler of Industry 4.0, the Open Platform Communication Unified Architecture (OPC UA) protocol [4]. The long chronological dispersion and duration of automation systems and the slow progress in research and applicability of modern protocols are still determining a widespread presence of legacy protocols. However, the industrial requirements determine the research of various wrapping solutions to increment performance [5]. In the near future, taking in consideration the increasing need for Ethernet-based communication procedures between various types of devices, it is expected that major architectures, established by the most efficient and popular technologies, will eventually interact at a large scale. One of the essential objectives for achieving fast results is the identification of the right solutions and the confirmation of their compatibility in authentic circumstances.

**Figure 1:**
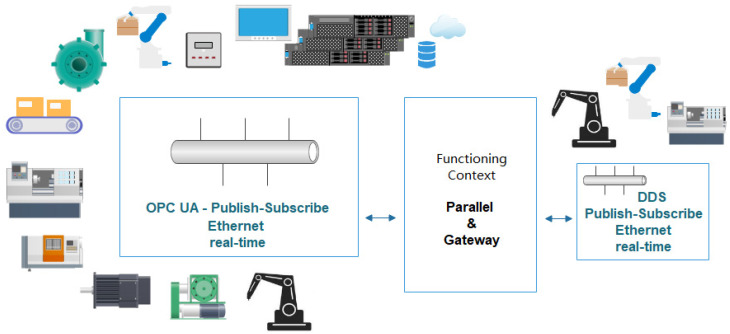
Schematic view of OPC UA—DDS protocol coexistence in the Industry 4.0 context.

The recent specifications of OPC UA [6] are allowing the protocol to evolve towards real-time industrial requirements. Although significant advances have been made [7,8], the protocol still has to reach its envisioned applicability. In this context, the research has to progress regarding the OPC UA Publish-Subscribe mechanism with a significant focus on real-time requirements. However, the production lines in the manufacturing industry include areas such as robots, co-bots, compact units where another emerging real-time oriented protocol will be certainly present in future architectures, and the Data Distribution Service (DDS) protocol defined by the Object Management Group (OMG) (see Figure 1). In this context, the coexistence analysis of OPC UA and DDS Publish-Subscribe solutions focused on real-time industrial requirements is essential. The current research proposes an analysis tool and methodology to evaluate the real-time functioning capability of OPC UA and DDS protocols, in parallel and in a gateway context.

There is another important aspect that must be further analyzed, concerning the real-time behavior for DDS and OPC UA. The industrial environment needs fast applicability. The current production lines contain mostly OPC UA client–server solutions and legacy protocols. The desire is to transition towards real-time oriented Publish-Subscribe protocols, and in this context, the infrastructure is also a critical issue (see [9]). Studies [10,11] are focusing on Time-Sensitive-Network (TSN) equipment, but the industry is requiring faster real-time constraint applicability approaches regarding operating systems and equipment, and not yet a complete transition towards TSN due to very slow progress of TSN within industrial usage.

In the above-mentioned context, the current work aims to:

- Define specific criteria that allow the examination of DDS and OPC UA in an unideal system, taking in consideration multiple challenges from the industry.
- Analyze the real-time behavior for DDS and OPC UA, implementing the necessary mechanisms for the process.
- Define an architecture that is suitable for parallel usage of DDS and OPC UA, that also offers the possibility for the two communication protocols to interact.
- Implement a DDS—OPC UA gateway application.

The following section addresses topics related to DDS and OPC UA in the IIoT and Industry 4.0 context together with real-time oriented infrastructure issues. The third section details the proposed architecture of the solution. The fourth section presents two case studies, including the obtained results, followed by the final section that further discusses the findings and concludes the research.

## 2. Materials and Methods

In the current chapter, the DDS and OPC UA protocols will be described from industrial and research perspectives, taking into consideration different contexts and applicability domains. Each of the four subchapters provides in-depth analysis and observations for the most important mechanisms and technologies that play an important role in time-deterministic communication scenarios. As from the implementation point of view, for the development of the current work, the eProsima Fast DDS [12] open-source software development kit (SDK) was used for all the implemented DDS entities, alongside the open62541 SDK [13] for all OPC UA implementations.

### 2.1. DDS in the IIoT Context

DDS targets the real-time behavior of the communication by implementing a publish-subscribe paradigm and also providing a request/reply paradigm for sending commands that expect an answer or for accessing individual services, assuring high efficiency for one-to-many, one-to-one, and many-to many scenarios. The availability of multiple mechanisms to be used for information exchange makes DDS a flexible and scalable solution when taking into consideration the constant evolution of the requirements in the context of IIoT. From the perspective of the publish-subscribe mechanism, the unidirectional exchange is done based on the Quality of Service (QoS) policies defined inside the architecture, allowing a set of multiple options from a developmental point of view. DDS implements a data-centric model, establishing the concept of a global data space which all the entities can access, a space where the information is propagated once the role of each entity is established. The publish-subscribe paradigm design is based on topics, and the receiving entities express the interest regarding a topic, and a match is made with the publishing entity of the desired topic. With a topic-based design, in complex architectures, the data exchange is simplified, allowing only the right data to arrive at the right subscriber, increasing the efficiency for real-time constraints and permitting a more abstract manner to categorize and encapsulate the data. It assures the scalability of the protocol in use cases where the architecture of the system contains many entities, and different types of data are published at different time recurrences. The entities can be grouped in domains, having isolated virtual spaces on where the publishers and subscribers can exchange specific data, improving the flexibility of the protocol and enhancing the volume and complexity of the data exchanged, making possible the development of more elaborated architectures.

As wire protocol, Real-Time Publish-Subscribe (RTPS) protocol is used, assuring interoperability over standard networks with emphasis on the real-time requirements. At transport level, RTPS can use TCP/UDP/IP (Transmission Control Protocol / User Datagram Protocol / Internet Protocol) providing access to all the DDS features alongside portability and compatibility with different DDS implementations. Similar to the DDS, the same principles regarding the association of the involved entities to a domain are applied to the RTPS as well, alongside the mapping of DDS subcomponents and features to RTPS. This allows to have an abstract way of dealing with specific details from system architectural perspective and roles definition for all the involved entities from wire level to above. Also, it maintains the flexibility towards different mechanisms, specific to the achievement of real-time requirements or related to discovery possibilities inside a domain.

For a communication protocol capable of achieving real-time responsiveness, the function calls must be executed in a context that is aware of the necessary time needed for the instructions to execute and the expected time based on application needs. Towards this principle, on Linux-based operating systems, DDS comes with the ability to provide a configurable blocking time-interval for functions that compete for resources. In cases where the configured blocking time-intervals are exceeded, there are measures in place that prioritize the most important operations, contributing in this way to the proficiency of the DDS protocol to comply with the development necessities for real-time applications.

Besides the split of the API in two layers, one specific to the wire protocol and another layer specific to more abstract DDS concepts, and besides the real-time behavior assured through build-in mechanisms, another important feature of the DDS protocol is represented by the discovery mechanisms. Simple discovery is based on the RTPS standard and allows compatibility with other DDS implementations. Static discovery implies the knowledge upon all Data Writers and Data Readers alongside data types and topics involved in the architecture, the entities identifying each other by specified IPs and ports. The discovery server mechanism, where the distributor of the discovery meta traffic information is represented by one of the existing entities belonging to the domain, constitutes another flexible solution in a centralized design.

With the evolution of complexity for the distributed architectures in the context of Industry 4.0, the systems are targeting the use of multiple protocols and devices with different types of requirements, making necessary the development of safety mechanisms at different layers for the prevention of system failures and fast recovery features that provide an increased robustness for the technologies involved in the industrial scenarios. For communication protocols that assure the interoperability between multiple devices that exchange high data volumes, the fast recovery of the communication procedures may represent the difference between a technology that is efficient in time-critical situations, capable of achieving hard real-time constraints, and a technology that is inadequate for time-sensitive operations. In the case of DDS, a mechanism for this type of scenario is depicted under the form of the Persistence Service. Based on the recovery process of a previous state of the system before shutdown, between the DataWriter component which is responsible for informing upon a modification of a data value specific to a topic, and the DataReader component in charge of consuming the data values, the Persistence Service allows for the storage of context-related details alongside the last notified change of the data values. Having this feature available, the system is able to restore the communication state previous to an unwanted shutdown and with the stored information as fast as possible. The involved entities responsible for the publish or subscribe operation can act accordingly to the situation, offering a clear view upon which data arrived before the stoppage event and which data need to be delivered at the exact moment of time after the recovery. These types of capabilities represent an advantage for all the IIoT technologies and provide, from a development perspective, a larger view and increased control possibilities upon the system. In the case of DDS, the Persistence Service increases the robustness and offers a high quality of service, making the technology suitable for a large variety of use cases with a wide range of requirements.

In the automotive domain, DDS is part of the AUTOSAR Adaptive Platform, with bindings to the Communication Management, alongside other acknowledged communication protocols over Ethernet, for example, SOME/IP. However, different from SOME/IP, which is universally used for intra-vehicle communication scenarios, DDS is considered to be used mainly for the manufacturing processes in the automotive field, being implemented in assembly and production lines for industrial robots. Additionally targeting the embedded systems and resource-constrained devices area, Micro-XRCE-DDS is available as a middleware solution that assures QoS and multi-platform support with guaranteed low resource consumption.

From the research perspective, DDS implementations are available for a variety of domains and use-cases. In the Ref. [14], the authors mention the advantages of the Publish/Subscribe paradigm alongside the adoption of RTPS as wire protocol for assuring interoperability between DDS implementations from different vendors. The solution is used for data exchanges among underwater vehicles, indicating the feasibility of DDS in extreme environments. In the Ref. [15], the challenges regarding the configuration of QoS for real-time systems are highlighted alongside the difficulty of implementing applications with high interoperability due to the lack of DDS standards, the consequences being an increased complexity in understanding DDS dynamic models. The authors provide a framework-based development tool for software reusability with different DDS implementations, useful in architectures with a large number of heterogeneous IoT devices.

### 2.2. DDS as ROS2 Middleware

The Robot Operating System 2 (ROS 2) is an open-source software framework which consists of algorithms, drivers, and tools for building complex robot applications compatible with various hardware equipment. It provides a communication system adapted for IIoT use cases and visualization and simulation mechanisms, helpful in architecture definitions and system behavioral observations at a low cost. With a whole ecosystem for development in place, ROS 2 gains popularity among both research and industrial fields [16,17]. The integration of other technologies, designed for obstacle detection, image processing, and dependency management between components is accessible, resulting in the rapid adoption of ROS2 as the desirable solution for the implementation of complex applications for distributed architectures specialized in robot control and odometry. From the perspective of communication middleware, DDS has been validated as a suitable solution for ROS 2 [18], providing decentralized communication possibilities between components and extending the capabilities towards the achievement of hard real-time requirements through the Publish/Subscribe paradigm.

As part of ROS 2, the default publication mode used by DDS is the asynchronous mode, meaning that the data are copied into a queue and the control over the publishing operation will be taken over by a different background thread. This thread will be in charge of consuming the data from the queue, leaving the main thread available even before the information is sent. With increased accessibility for the initiation of the publishing process, this mode is targeted for nodes that do not have rapid time expectancies and are not in charge of time-sensitive operations. The second mode that is available is the synchronous publication mode, where the control over the publishing operation is owned by the main thread. No other functions are executed before the data are sent due to certain mechanisms in place that prevent other parts of the application from interfering with the main thread. The advantages of the synchronous mode are related to better control over necessary time-intervals in which the data are expected to be sent, and where higher data volumes with minimal latency are delivered to system nodes in charge of time-critical events. The configuration between the two modes is attainable with ease, confirming the flexibility granted by DDS for complex systems with various demands.

The inclusion of ROS 2 in academic projects is in a beginning state. The main obstacle is represented by the difficulty of migration from ROS 1 due to multiple requirements in terms of technological updates, a consequence being the reduced usability of existing developments, as stated in the Ref. [19]. However, it is expected for ROS 2 to generally replace the previous version because of the new features that are suitable for the evolving requirements present in Industry 4.0 scenarios. DDS can be viewed as a main contributor to the advancement and expansion of feasibility from the communication perspective. In the Ref. [20], the importance of DDS as part of ROS2 is detailed alongside the importance of different DDS implementations for achieving the major objectives of ROS2 concerning the multiple robots cooperation, the usage of smaller embedded systems with limited resources, the real-time constraints that are present in multiple industrial use cases, and the communication over unstable networks. The authors propose a solution for dynamically binding proper DDS implementation, suited for specific situations, as a middleware to ROS2. It is expected for DDS to enhance and adapt ROS2 as a solution that is compatible with vast industrial control architectures, establishing a footprint as an efficient communication technology with high potential.

### 2.3. OPC UA: Established in Industrial and Research Circumstances

The OPC UA protocol is present in industrial automation domains as the main technology capable of offering a wide variety of features on all the levels of the OSI model (Open Systems Interconnection), being capable of overcoming most of the growing demands of the industry. It implements the classic Server/Client paradigm, having a dominant applicability at the moment in multiple fields, like the Water Industry, Manufacturing, Power Industry, and others. More recently, it has offered the Publish/Subscribe paradigm as a response to the growing real-time requirements existing in the Industry 4.0 advancements. From a research perspective, OPC UA represents a major topic for the academic and industrial domains. Studies have been made [21,22] focusing on multiple features provided by the protocol, and the results lead to the establishment of OPC UA as a significant technology in the industry. With the recent specifications [5] that defined the Publish/Subscribe mechanism, new studies have been conducted involving comparisons between the new capabilities and the expanding requirements.

In the Refs. [23,24], OPC UA was implemented in the context of car-to-infrastructure communication, proving the feasibility of the protocol in use-cases where interaction between the automotive and automation domains is necessary. In the Ref. [25], the authors implemented a prototype integration of the OPC UA and TSN technologies in the AUTOSAR Adaptive standard. In the Ref. [7], the OPC UA Publish/Subscribe mechanism is analyzed in detail and compared to other similar mechanisms from other communication protocols, and architectural improvements are suggested for specific industrial use-cases. In the paper by the Ref. [26], the applicability of the OPC UA protocol was extended towards image transmission scenarios, implementing multiple UDP Publish/Subscribe channel transmission. The various use-cases in which OPC UA was implemented with significant results indicates the robustness and the flexibility that propagated the protocol as an important technology present in the industry. With the addition of the Publish/Subscribe mechanism, the increased capabilities have expanded its relevance to other areas. In the context of the expanding real-time requirements associated with Industry 4.0, OPC UA offers a high quality of service, and it is expected to continue to extend and improve for an accessible adaptation to any IIoT necessity.

### 2.4. TSN: Evolution, Challenges and Expectations

When discussing the most promising communication protocols over Ethernet for distributed architectures, the TSN technology must be mentioned as it plays an important role in all use-cases where real-time assurances are needed at network level. As a set of standards and mechanisms that are configurable for achieving different objectives, TSN adoption can raise numerous challenges for any communication structure. One of the obstacles is related to the large number of interdependencies that the TSN implies, lots of adjustments being needed for all the devices and applications involved, generating a significant effort from a development perspective. Another obstacle is represented by the configuration possibilities available for each standard, making the evaluation of the right set-up a complex process and generating scalability issues. A standard configuration scheme is a desirable step to achieve in the near future, however it is a difficult task, taking in consideration the parameters that could influence such a procedure (the number of devices, the capabilities of each device, the most efficient set of mechanisms needed for a certain scenario, real-time requirements, security and safety limitations, etc.). The most efficient approach, for the moment, adopted in the industry at first implies a correct identification of the necessary TSN mechanisms for the desired use-case and subsequently configures each mechanism, increasing the optimization of the system in gradual steps.

Even with considerable benefits provided by TSN, slow progress can be seen in both research and industrial usage. The main reason for this is represented by the limited amount of devices that support TSN technology, found on the market. However, the specialized hardware companies are starting to focus on the development of devices suitable for IIoT use-cases with real-time requirements, that implement specific TSN standards at the data link layer. A relevant example can be observed in the automotive domain, where controller-to-controller Ethernet communication scenarios become more frequent and hardware equipment has to deal with the already established time-constricted operations in a synchronized manner. At the moment, the time guarantees of the networks are desirable for enhanced performance of the IIoT systems, and even with all the challenges associated with the transition to TSN technology, the evolution towards improved communication infrastructures is essential for the industry.

## 3. Architecture

The current work focuses on two major communication protocols, DDS and OPC UA, specialized in Ethernet communication, adapted for a wide range of applications, and capable of satisfying real-time and synchronization requirements, highly required for the large-scale systems that are expected to progress towards the next connectivity level. Even with similarities regarding the capabilities, the implemented mechanisms, and the component technologies, an important differentiation can be observed regarding their current purpose. OPC UA is more present in factory and industrial automation, recently assuring robust controller-to-controller communication, and DDS is more present in multi-robot control and cooperation procedures through its adoption as middleware in ROS2. With the expected evolution towards IIoT and Industry 4.0 and with the rapid expansion to multiple industrial fields, the probability of major technologies like OPC UA and DDS to collide and cooperate becomes quite high.

**Figure 2:**
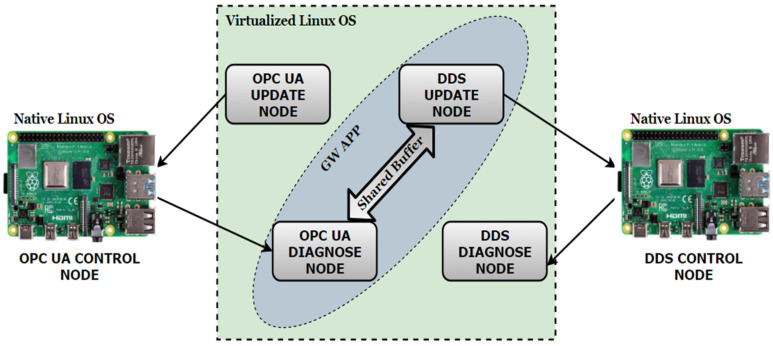
System architecture.

To create a probable next-step scenario and to apply multiple approaches that address ongoing concerns from the industry, a multi-node mirror architecture for the two protocols was defined, with three communication nodes for DDS and another three nodes for OPC UA. Each node has a specific role, running on different devices and each type of device running the application on a native or virtualized general-user Linux-based operating system. The defined architecture was meant to comply with both performance comparison scenarios, and also with interaction scenarios over a gateway application, with the purpose of confirming the compatibility and feasibility between DDS and OPC UA alongside a general definition of criteria and expectations from an architectural point of view. When adopting or selecting a technology for certain use-cases, especially with cutting-edge technologies, the development process is slowed down by numerous uncertainties generated by undocumented challenges. Rather than applying the classic trial-and-error strategy, the research community has to provide guidelines, performance criteria, architectural perspectives, and point out advantages and disadvantages in a neutral manner, shaping a comprehensive view of the possibilities. The system architecture can be observed in Figure 2.

The OPC UA and DDS control nodes are defined for control simulation purposes, and they are intended to be the main consumers of the data. The control nodes run on Raspberry Pi 4 devices with a native Linux-based operating system. They must be viewed as the primary control segment (for example: control of a PLC device, a robot control node, or a production line control entity) of the architecture in a real-life scenario. Both are designed with two communication subcomponents:

- A subscribe component which receives updated information from the update node at different time recurrences;
- A publisher component which sends the information to a diagnosable node, for possible diagnostics or safety operations specific to a certain industrial process.

The update nodes are the main producers and distributors of the data. They support complex configuration options, being accessible only by the control nodes.

The diagnosable nodes receive data only from the control nodes, and can perform check operations for the distributed data or serve as a gateway component, allowing the interaction between OPC UA and DDS for complex control scenarios (for example: sending data from a PLC device controlled by an OPC UA control node to a motor operated by the DDS control node). When the system is configured for gateway use-cases, the complementary entities of different protocols (OPC UA diagnose node—DDS update node and vice versa) exchange information through a designated shared buffer. The system architecture offers high flexibility depending on the requirements, allows the observation of each operation for different real-time expectation, and supports multiple control situations with minimal efforts at configuration.

### Comparison to Related Work

Other studies have been performed with the current technologies in a comparison context, with significantly different approaches, objectives, and achievements. In the work by the Ref. [27], the authors evaluated and compared performances of OPC UA and DDS alongside other significant communication protocols, presenting the round-trip time based on the packets’ sizes in different stress scenarios.

The current distributed architecture contains multiple entities and involved routes, and develops specialized nodes with different purposes, implementing multithreading applications for all the nodes, and also oversees the reaction of the general-user Linux-based operating systems. In other words, the current work does not only evaluate the communication protocols, but rather the interaction, efficiency, and usability in IIoT scenarios with expected specific preconditions, such as multiple devices, operating systems with different particularities, unideal networks, and different real-time demands. Such evaluations oriented on concrete use-cases and industrial processes can prove useful when considering the adoption of a technology for a certain domain. The research community has the potential to be the principal enabler for the evolution of current industrial implementations, architectures, standards, and requirements towards the Industry 4.0 and IIoT.

Another significant difference from the work by the Ref. [27] is represented by the evaluation criteria established for the current work. The first criterion implies:

- The individual testing of the real-time responsiveness of each operation (publish, subscribe) at device level;
- The comparison with the ideal expected results.

The distributed architecture and the present operating systems on each node are key factors in the evaluation. A main objective at this point is represented by the obtained percentage-based results that offer the estimation possibilities in real-life industrial scenarios, where each operation from each level of the architecture has an established real-time expectation. A second objective is related to the similarities and differences for OPC UA and DDS in identical architectural scenarios, with close percentage-based results for different time intervals, highlighting the compatibility of co-usage in future industrial architectures with far from ideal preconditions and set-up possibilities, as it is expected in the slow progression to IIoT.

The second criterion implies the data buffering of the received information (the results from the subscribe operation) comparing the results with the expected amount of received values. The objective is not only the overseeing of the operation (publish, subscribe) execution, but also the acknowledgement of received data, with the network stability playing an important part in the outcome.

In the work by the Ref. [28], the authors converted Message Queuing Telemetry Transport (MQTT) and Advanced Message Queuing Protocol (AMQP) messages generated by an OPC UA publisher into DDS messages that are exchanged between a DDS publisher and subscriber. The major differences present in the current implementation are related to real-time behavior and architectural perspectives. MQTT and AMQP are optional transport protocols for OPC UA, and both work in a broker-based design. The transport protocol used for the current development is UA Datagram Protocol (UADP), a transport defined by the OPC Foundation for use-cases that require UDP communication and IP multicast possibilities. The UADP transport solution offers direct connection between the OPC UA publishers and subscribers through the network infrastructure, offering synchronization possibilities between the host devices. Different from the broker-based design, the direct connection approach requires a complete OPC UA code understanding (on all the levels of the OSI model) from all entities, as the DDS protocol also requires. The broker application itself is considered additional software, and its functionality (the distribution of the messages) is considered out of the scope of the specification, as stated in [6], being an MQTT/AMQP communication application as for any other MQTT/AMQP provider and consumer (without the need to understand the OPC UA code). The broker-based design is specific to high-latency scenarios (usually interaction with Cloud architectures) and in the case of OPC UA communication, “the OPC UA Publisher and the OPC UA Subscriber are messaging clients” [6]. For the current gateway application, all the involved entities and components use a direct connection approach providing a high level of time determinism for all the procedures, and where all the developed components have complete understanding of DDS and OPC UA-specific codes, offering complete information exchanges between OPC UA publishers and OPC UA subscribers/subscriber components (not MQTT/AMQP transport level entities) and the data are exchanged between different technological endpoints through an intermediary node (the gateway application) using the OPC-defined way of distributing the messages, assuring low latency, additional security, and using the network infrastructure for Ethernet-based communication.

## 4. Case Study and Results

The current work focuses on two case studies. The first one targets observations on how each communication protocol reacts in accordance with the operating system for operations executed at different time recurrences. It analyzes how efficiently the technologies behave when running on operating systems without enhanced real-time capabilities and using a network that does not offer time guarantees for packet transmission. The second case study proposes a gateway solution for scenarios where protocol interaction is required. The switch between the two use cases can be performed with little effort at configuration and no architectural changes.

### 4.1. Case Study 1

The development of applications for multiple device communication set-ups must assure high efficiency and good resource management on all devices. For the current case study, the Publish-Subscribe mechanism was used for both communication protocols, targeting real-time behavior at the application level for all nodes.

The initialization of DDS communication is done through a series of similar steps on all devices. First, on each side, a participant is initialized with domain-specific information. A participant is an entity (which can either be a publisher or a subscriber) and can exchange data inside the domain. Once the participant is configured, it must be added to the domain. The second step is related to the role of each participant. The role is selected through predefined data types, specific to either a publisher or a subscriber that need a certain configuration based on particular attributes. After the configuration is finished, the role is assigned to the participant. The role itself could be perceived as a subcomponent of the participant from a software perspective. The third step contains multiple configurations related to the network, available topics, or matching operations between reader and writer entities depending on the use case.

The OPC UA data exchange is initialized in a similar manner to the implementation in work by the Refs. [7,24]. For the development of the publisher entity, the publish–subscribe connection is initially realized, and afterwards, the data set is defined and the payload assigned to the data field. The last step is the configuration of the writer group and the data set writer. For the subscriber part, after the publish–subscribe connection is initialized, message decoding, filtering, and data extraction is performed on the recurrent arriving OPC UA PubSub messages.

**Figure 3:**
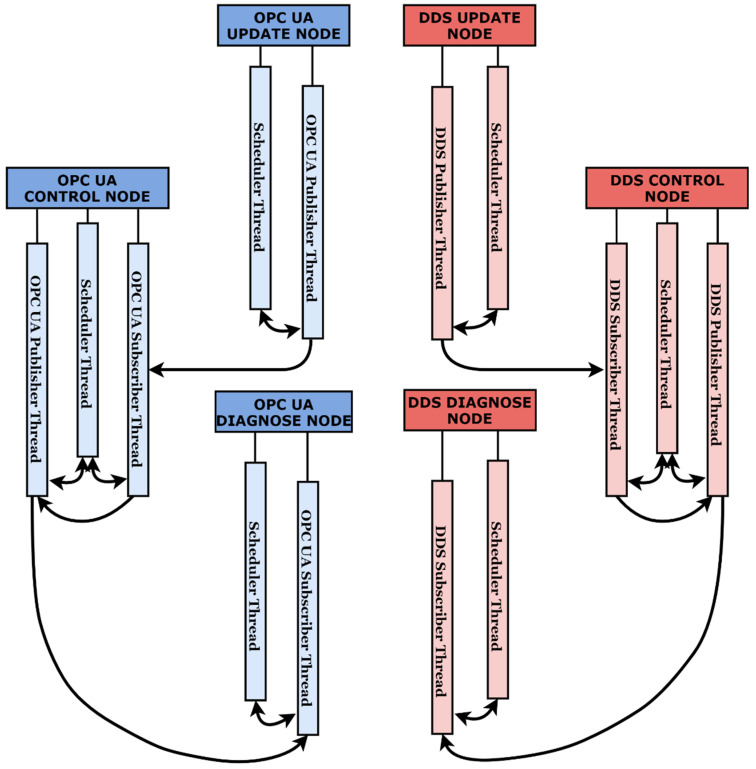
Multithreading nodes from an architectural perspective.

The main objective of the current case study is represented by the behavior analysis of DDS and OPC UA protocols in a similar architecture, with similar devices and operating systems used, on identical real-time driven scenarios. To better observe the behavior and better quantify the responses of the technologies to real-time demands, the authors defined two major evaluation criteria. The criteria are not necessarily meant to state that one technology is better than the other, but rather to establish that the responses are not hugely different, and assuring that the interaction between OPC UA and DDS is possible and can add value to cross-domain architectures, expected to use both protocols in the future. With general-user Linux-operating systems which do not have enhanced real-time capabilities, and without using real-time Ethernet technologies like TSN, the performance in real-time scenarios is expected to decline for under 10 ms expectations between data exchanges. The idea of the current case study is to showcase in a quantifiable manner the declining from 10 ms expectancies to 5, 2, and 1 ms expectancies for both protocols. The first criterion is represented by the function calls that the application can run once the time intervals become shorter. For example, if the application has set a publishing time interval of 5 ms, the result will compare the actual number of function calls that happened in 10 ms (a time interval still guaranteed to deliver by the operating system) against the expected results (for 5 ms, the expected publishing operation call result is 2 in 10 ms). The results will be measured by a custom scheduler developed for this case study, which will run on a different thread from the communication operations. The result is not influenced by the time interval set for the publishing or subscribing procedure. The accuracy of the scheduler is verified through a recurrent signal generation at a certain time interval, the signal being measured by an oscilloscope, guaranteeing the accuracy of the scheduler. In rare cases where desynchronizations appeared, the results were not taken into consideration and the test would be restarted. The scheduler runs on all devices as a different thread. The designated check functions that compare the actual calls for the communication procedures and the expected results create a detailed log for each iteration of a scenario, providing the ability to identify both network and scheduler desynchronizations for the entire architecture. Having multiple nodes on different devices, all node applications are multithreading and the number of threads is increased for the control nodes that have both subscriber and publisher roles assigned. A better view upon each of the multithreading node applications can be observed in Figure 3.

With a scheduler component designated for the real-time behavior and multiple check functions for all established time intervals to be observed (10 ms, 5 ms, 2 ms, 1 ms) in place, the first criterion of the case study can be covered and the results can be quantified as percentages for each node.

The second criterion is represented by the data acquisition analysis on each receiving node within the same previously defined time intervals. An implemented data-buffering mechanism was designed to store all the arriving information based on the configuration. At the end of the process, the data-buffering mechanism generates a log file where all the received values can be observed and compared to the number of expected values. Numerical percentage results are produced, allowing to identify network and device desynchronizations, and also providing a possible safety mechanism for elaborate scenarios. The different nature of the operating systems present on each device allows a comparison between similar procedures, with the diagnosable nodes serving as a last consumer entity on the architecture, permitting better monitoring of the final result of data distribution among three different nodes. The data-buffering mechanism uses configurable buffers, assuring high flexibility for any desired data type and resource efficiency, being used for data acquisition by the communication thread and obtaining the time source from the scheduler component. It can be perceived as a multithread mechanism for all nodes that support the subscriber role.

Having results from both perspectives, from the function calls verification and from the data buffering mechanism, in-depth monitoring is possible for the OPC UA and DDS protocols’ response on a multi-node architecture. The particularities and set-up of the system assure a context close to the current general state for the adoption of the targeted technologies in IIoT scenarios. Multiple challenges of the industrial field are taken in consideration, highlighting the many positive and negative aspects that impact the Industry 4.0 advancement.

### 4.2. Results after Case Study 1

The implementation of Case Study 1 alongside the development of the custom necessary surveillance mechanisms and the development of the communication infrastructure on all devices was successful. It proves the feasibility of the architecture and provides quantifiable real-time results for all the nodes, in accordance with the two above-mentioned criteria. A considerable number of tests were performed on all the nodes to minimize the relativity of the behavior due to the particularities of the system, the unpredictability of the operating systems in hard real-time situations, and the possibility of device desynchronization over the network.

The results for the functions call verification process can be observed in Table 1, Table 2, Table 3 and Table 4 for DDS, and in Table 5, Table 6, Table 7 and Table 8 for OPC UA for the first criteria of the case study. The Tables present success rates for each time recurrence.

From the DDS perspective, as can be seen in Table 1, Table 2, Table 3 and Table 4, the implementation assures that data can be exchanged lower that 10 ms, on both types of operating systems, and even at 5 ms, the data are distributed efficiently, with a negligible but existent failure possibility. Based on the numbers, the discussion for time intervals between 10 ms and 5 ms should focus on how tolerant the system can be in a publish–subscribe exchange where the volumes and speeds of the distributed data are expected to vary. From the OS standpoint, the experiment proved that native Linux-based operating systems provide increased efficiency and are a more stable option, with less probabilities for delays and desynchronizations, the difference being quantified. From the roles perspective, the Publisher operation performed better than the Subscribe operation on both the operating systems, in the current unideal conditions. However, this could prove unimportant in ideal cases where for both roles, the exchange is managed by additional mechanisms. For time intervals below 5 ms, the fail probability cannot be disregarded, making the accomplishment of the real-time requirements not possible in the current system. For the 1 ms and 2 ms intervals, the numbers are very close from one type of OS to another, confirming that, from a real-time perspective, there are too many improbabilities to exchange data at such recurrences without special mechanisms in place.

The OPC UA implementation also confirms that the communication is successful between all the nodes, the results being improved on the devices with native Linux-based OS, with still 100% recurrent function executions at 5 ms. For intervals under 5 ms, even for the devices that operate with a native OS, the probability of failure is high and confirms the necessity for technologies like TSN and other mechanisms to guarantee data exchange at recurrences under 5 ms. The difference between the DDS behavior in the same circumstances is not significant for both protocols, being not feasible to increase efficiency in the current context. From the role perspective, the subscribe procedure performs better under 5 ms. However, implementations such as in the Ref. [7] are proposing a different approach meant to increase the efficiency of the publish and subscribe operations, although such a design impacts the whole architecture. Based on the results of the recurrent function call verification in identical set-ups, both communication protocols have close behaviors in real-time scenarios, being capable of exchanging data even without designated integrated mechanisms, and both DDS and OPC UA can be considered as highly proficient and flexible solutions for IIoT applications.

**Figure 4:**
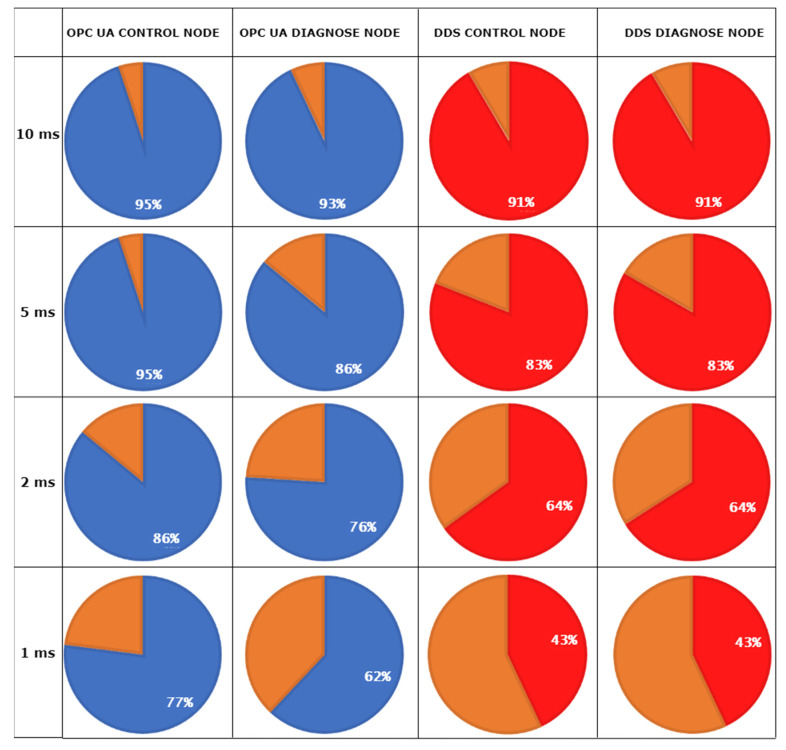
Data-buffering success rate percent-based results.

The second criteria of Case Study 1 focuses on the data-buffering mechanism designated to highlight how OS and device desynchronization influence the receiving data, offering statistical results at different time intervals. The results can be observed in Figure 4.

The result for the data-buffering process on the Subscribers side confirms the results obtained from the recurrent function call verification. The numbers are proportional for both criteria, with expected decreased percentage values for the data-buffering process, validating the additional impact that the network stability has for the data exchange procedure, over the already existent impact generated from OS real-time limitations. Although at under 5 ms intervals, the differences between the protocols seems to slightly increase, there are still uncertainties regarding how much the interaction and data exchange between the protocols can be affected, a gateway scenario being of interest in this case.

### 4.3. Case Study 2

For Case Study 2, the main objective was the implementation of a gateway application for the current architecture, offering the possibility to acknowledge the feasibility of DDS and OPC UA in IIoT multi-device scenarios. Having a gateway application for cross-domain control and data exchange operations through Ethernet technologies confirms the high capabilities of two well-established communication protocols and expands the possibilities towards future advancements. The impact of Ethernet protocols in hard real-time use cases is difficult to estimate. Even if there are numerous solutions available and compliant with real-time demands, the general understanding must not focus on how fast a communication protocol is able to deliver data, but rather, how stable the delivery is, at what time intervals the exchange can fail, and what are the factors that can influence the performance of the chosen protocol. The current development suggests a detailed exemplification of how the above-described impact can be observed and understood even in scenarios where the data are delivered at fast recurrences.

The industrial context of the OPC UA—DDS gateway application is highlighted using the current architecture and proposing for the OPC UA and DDS control nodes to simulate industrial processes that are specific to automation and robot control domains. In the case of an OPC UA control node, the information arrives from the OPC UA Update Node and logical operations are performed to obtain two different types of payloads, simulating inputs that could be taken from a PLC device. The two types of messages are sent alternately to the gateway application, which serves as an OPC UA subscriber to the control node and as a DDS publisher to the DDS control node. Once the message is received by the DDS control node, a digital signal is generated accordingly to the type of the received message (corresponding to high or low states) simulating the control of an actuator that requires fast recurrent pulses. The generated digital signal is monitored using an oscilloscope. Thus, for each time interval set for data exchange between nodes, the signal is measured, offering the possibility to confirm the length and frequency of the pulses, meaning that the whole propagation of the data through the architecture can be measured. The expected desynchronizations and stability problems that may occur for under 10 ms intervals can be visualized with ease, and the qualitative degradation of the signal can be observed in a significant manner. This provides a suggestive perspective upon the necessity for additional technologies capable of guaranteeing the time synchronization between devices and network stability in IIoT use cases. The gateway application facilitates the propagation of the data between multiple nodes, devices, and distinct protocols and operating systems without different configuration needed for the already implemented software. The solution confirms that both OPC UA and DDS are compatible and feasible for such scenarios, even in unideal systems, increasing the potential towards more complex and scalable applications for multiple industrial domains.

### 4.4. Results after Case Study 2

**Figure 5:**
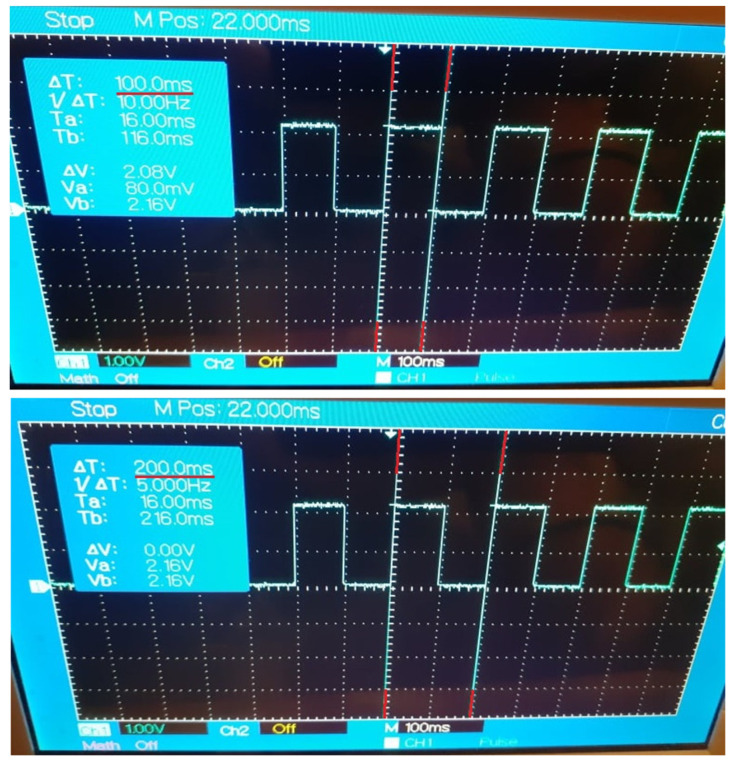
Generated Digital Signal based on payload delivered by the Gateway Application at 100 ms recurrence.

**Figure 6:**
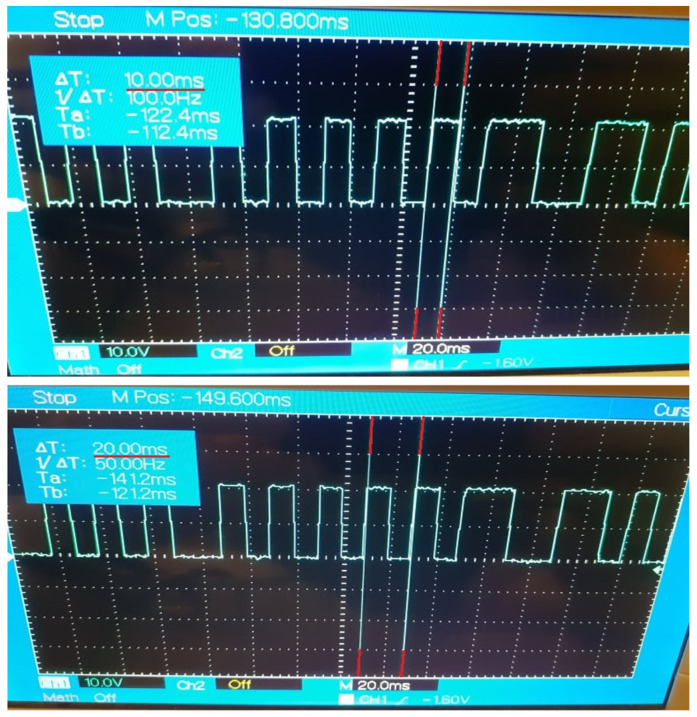
Generated Digital Signal based on payload delivered by the Gateway Application at 10 ms recurrence.

**Figure 7:**
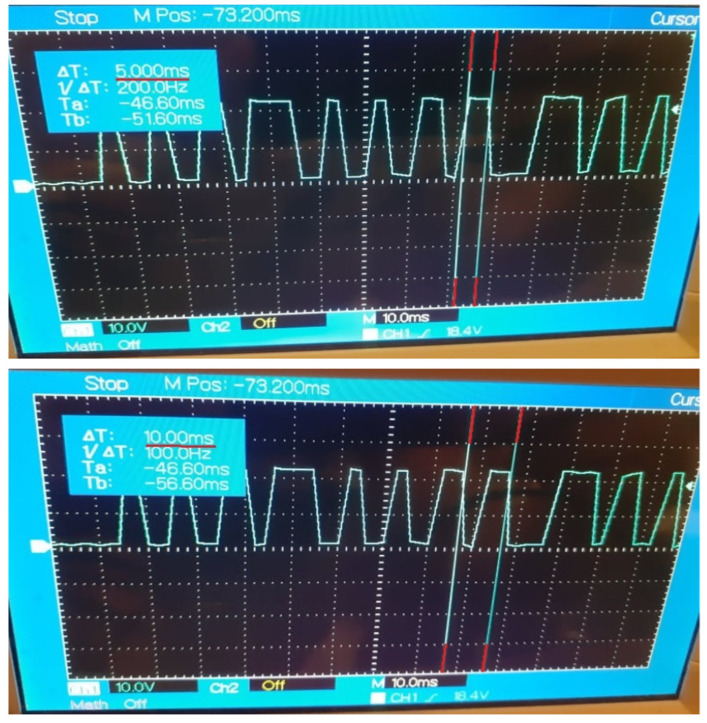
Generated Digital Signal based on payload delivered by the Gateway Application at 5 ms recurrence.

**Figure 8:**
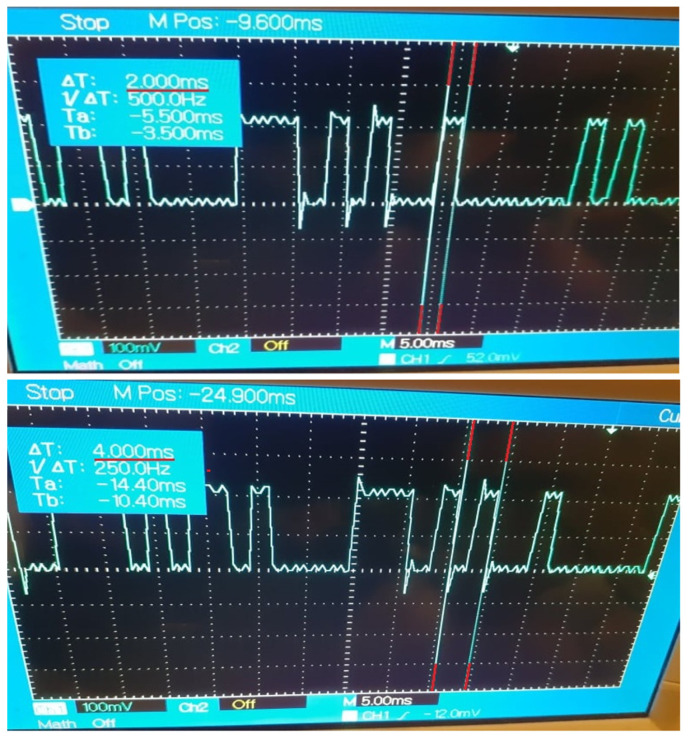
Generated Digital Signal based on payload delivered by the Gateway Application at 2 ms recurrence.

**Figure 9:**
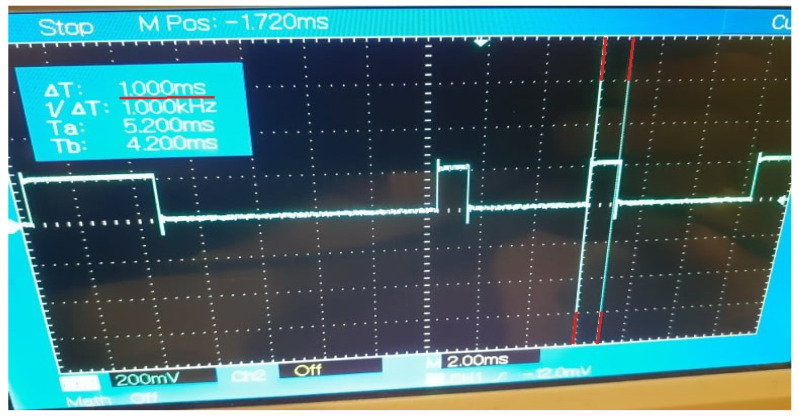
Generated Digital Signal based on payload delivered by the Gateway Application at 1 ms recurrence.

The implementation of Case Study 2 was successful. The gateway application facilitates the data exchange between the control nodes at configurable recurrences and the results are observable and measurable, highlighting the impact of device desynchronizations and network instability. The current results are in concordance with the results of Case Study 1 that estimated a decrease of efficiency for the data delivery, with each transmission performed at a faster cycle. The evolution of the data propagation through the multi-node architecture is noticeable through the digital signal generated by the DDS control node based on the specific payload received from the OPC UA control node. The outcome of Case Study 2 can be observed in Figure 5, Figure 6, Figure 7, Figure 8 and Figure 9 for different recurrences.

In Figure 5 (100 ms), the signal is generated in the expected form and with the expected frequency, meaning that the data arrived accordingly. The network is stable enough to avoid desynchronizations between the transmissions, and the data exchanged between all nodes are delivered with high efficiency. The potential unfavorable factors were not impacting the process.

In Figure 6 (10 ms) the length of the signal is correct, and the cycles tend to have the desired regularity. However, slight desynchronizations can be detected at random times. meaning that some messages are not received in time by the control node, the main cause being the inability of the network to guarantee real-time deliveries in this case.

In Figure 7, Figure 8 and Figure 9, for all intervals under 10 ms, the observable results prove the capability of the protocols and the gateway application to deliver data at the desired intervals. The disadvantages for such fast exchanges are represented by the additional factors besides the network stability, factors like the delayed response of the operating systems of multiple nodes, as the generated signal is the outcome of multiple nodes exchanges and where each delay from any involved node can perturb the payload delivery. With each additional delay, the risk for inaccurate deliveries increases, the spikes of the signal being irregular as the speed of the transmissions grows. The reliability of the communication protocols in the current system declines in concordance with the results of Case Study 1.

## 5. Discussion and Conclusions

With the proposed architecture and concept, respectively with the case studies implemented proficiently, and based on the obtained results, the following objectives of the current work have been achieved:

- The two defined criteria for the examination of DDS and OPC UA behavior provided a complex perspective towards the capabilities of the selected protocols in a system that considers current challenges specific to multi-device communication over the Ethernet. The potential of the current criteria can extend to future developments that address specific improvement steps, or can be adapted to multiple particular systems and technologies with similar goals.
- The implemented mechanisms used to analyze the real-time behavior of DDS and OPC UA confirmed a high level of efficiency, and the obtained quantifiable results expand the current perception regarding the targeted technologies to new industrial and research areas.
- The defined architecture has proved to be reliable for both common and parallel usage of the protocols, delivering the desired level of flexibility and scalability. The diversity of industrial factors that disfavor the ideal responses from DDS and OPC UA adds authenticity to the experiment and allows the adoption of similar architectural concerns to a wide range of applications.
- The development of the OPC UA—DDS gateway application expands the applicability of the protocols to cross domain scenarios, reconfirms the feasibility and high quality of service claims for both technologies and in the current context, it offers a practical viewpoint concerning compliance to real-time requirements.

The general approach to Industry 4.0 emphasizes on large data volumes exchanged between different types of devices in real time, targeting the interaction between large systems with different capabilities and expectations. The technologies are expanding rapidly and the complexity of the requirements grows in a proportional manner. The current studies and advancements need to establish clear directions in terms of implementation and evaluation practices, considering the current obstacles and anticipating future unavoidable challenges. The applicability of the communication protocols must extend by continuously taking in consideration the advantages and disadvantages provided by each one, and the research field is essential to find ways to increase the efficiency and to define explicit steps for overcoming the unfavorable factors that are present in IIoT use cases.

The capabilities of the communication protocols can significantly be improved in systems that assure a higher real-time response capacity. However, for such situations, the adoption of additional software and hardware technologies and the usage of specially designed operating systems is mandatory, increasing the complexity of establishing a set of infrastructural circumstances (hardware equipment, multiple software components implementations, complex software integration methods, high configuration needs, automation testing possibilities) for maximized technological efficiency and cost effectiveness.

The relevance of the strategy and the conclusions imply to underline future possible improvements and perspectives regarding the presented concepts, alongside challenges surpassed during the development of the current work. The current work provides significant insights to the current concerns of the industrial and academic domains in the attempt to progress to Industry 4.0 principles. Future steps can be considered for maximizing the efficiency of DDS and OPC UA in the IIoT context and for defining additional methods of analysis and development of complex applications with diverse requirements. A first step in the process could be represented by the adoption of Linux-based operating systems adapted to hard real-time situations for all devices alongside the integration of the already implemented analysis infrastructure. A second step could imply the adoption of software and hardware mechanisms capable to overcome the network instability problems, specific to the TSN topic. After the modular inclusion of each additional improvement, for each stage of the conversion to an ideal system, the obtained results need to be compared and synthetized, for a better-crafted perspective upon how every contributing factor impacts the architecture and the performance of all the technological components. With the potential of the communication protocols attested and still expanding on industrial and research level, future experiments can contribute considerably to the Industry 4.0 evolvement.

From the implementation point of view, the main challenges of the current project are related to the multithreading design for each application distributed across multiple devices. Having on each device multiple threads that are managing separated procedures, the protocol specific configurations and the synchronization of the threads had to be done with care, taking in consideration the real-time scenarios desired to be evaluated. The development of the mechanisms corresponding to Case Study 1 criteria represented a difficult step, targeting a high degree of applicability and reusability. As for the gateway application use case, the main impediment was the successful integration of different software parts specific to different protocols, a common struggle that is also present in the industrial context for state-of-the-art technologies. From the evaluation perspective, the set-up of all the entities for the various stages of the process required consistent effort, alongside the high number of tests necessary to be performed for generating the results. From the conceptualization point of view, the identification of significant, decisive criteria that can also be applied at a larger scale represented a major challenge, together with the establishment of an architecture capable of addressing diverse topics and suitable for all case studies.

The applicability of the current study is certain in the manufacturing industry, providing the tools, mechanisms, and results regarding OPC UA and DDS protocol coexistence analysis focused on real-time functioning and fast applicability considering non-ideal infrastructure. Additionally, the study depicts a methodological strategy for the protocol of real-time coexistence analysis that can be used by the research community.

## Figures and Tables

**Table 1:** DDS Update Node—Virtualized Linux OS—Publish Operation.

Publish Operation—Recurrent Execution Check			
10 ms	5 ms	2 ms	1 ms
≈100%	≈90%	≈74%	≈64%
TOTAL Number of Tests: 2790			

**Table 2:** DDS Control Node—Native Linux OS—Publish Operation.

Publish Operation—Recurrent Execution Check			
10 ms	5 ms	2 ms	1 ms
≈100%	≈93%	≈84.6%	≈77%
TOTAL Number of Tests: 2865			

**Table 3:** DDS Control Node—Native Linux OS—Subscribe Operation.

Subscribe Operation—Recurrent Execution Check			
10 ms	5 ms	2 ms	1 ms
≈100%	≈85%	≈65%	≈48.5%
TOTAL Number of Tests: 2805			

**Table 4:** DDS Diagnose Node—Virtualized Linux OS—Subscribe Operation.

Subscribe Operation—Recurrent Execution Check			
10 ms	5 ms	2 ms	1 ms
≈100%	≈85%	≈65%	≈47%
TOTAL Number of Tests: 3015			

**Table 5:** OPC UA Update Node—Virtualized Linux OS—Publish Operation.

Publish Operation—Recurrent Execution Check			
10 ms	5 ms	2 ms	1 ms
≈100%	≈95%	≈81.2%	≈56%
TOTAL Number of Tests: 2685			

**Table 6:** OPC UA Control Node—Native Linux OS—Publish Operation.

Publish Operation—Recurrent Execution Check			
10 ms	5 ms	2 ms	1 ms
≈100%	≈100%	≈87%	≈56%
TOTAL Number of Tests: 2970			

**Table 7:** OPC UA Control Node—Native Linux OS—Subscribe Operation.

Subscribe Operation—Recurrent Execution Check			
10 ms	5 ms	2 ms	1 ms
≈100%	≈100%	≈91%	≈85%
TOTAL Number of Tests: 3015			

**Table 8:** OPC UA Diagnose Node—Virtualized Linux OS—Subscribe Operation.

Subscribe Operation—Recurrent Execution Check			
10 ms	5 ms	2 ms	1 ms
≈100%	≈87.5%	≈77%	≈64%
TOTAL Number of Tests: 3015			

## Data Availability

Not applicable.
